# Using Marketing Science to Understand Contraceptive Demand in High‐Fertility Niger

**DOI:** 10.1111/sifp.12078

**Published:** 2018-11-26

**Authors:** Sarah L. Dalglish, Jessica Vandermark, Clémentine Rossier, Adama Kemou, Hope Neighbor

## Abstract

Global initiatives aim to add 120 million new family planning (FP) users by 2020; however supply‐side interventions may be reaching the limits of their effectiveness in some settings. Our case study in Niger used demand analysis techniques from marketing science. We performed a representative survey (N = 2,004) on women's FP knowledge, attitudes, needs, and behaviors, then used latent class analysis to produce a segmentation of women based on their responses. We found that Nigerien women's demand for modern FP methods was low, with majorities aware of modern methods but much smaller proportions considering use, trying modern methods, or using one consistently. We identified five subgroups of women with distinct, internally coherent profiles regarding FP needs, attitudes, and usage patterns, who faced different barriers to adopting or using modern FP. Serving subgroups of women based on needs, values, and underlying beliefs may help more effectively drive a shift in FP behavior.

High‐quality family planning (FP) programming, in addition to supporting women's control over their bodies, relationships, and family size, is one of the most cost‐effective investments for reducing maternal and neonatal mortality, preventing stillbirth, and reducing disabilities (Cleland et al. 2006; Black et al. 2015). International partnerships aim to reach 120 million additional women with FP services by 2020 in the world's poorest countries, and ultimately provide universal access (Fabic et al. 2015). Many FP interventions have focused on supply‐side programming to ensure contraceptive availability and ease of access. However, understanding demand‐side determinants of FP use is particularly critical in sub‐Saharan Africa, where fertility levels have not declined as in other regions, and unmet need remains high in spite of rising knowledge of and access to modern methods (Casterline and El‐Zeini 2014; Cleland, Harbison, and Shah 2014). This is also the region with the highest rates of overlapping sexual and reproductive health risks (Schelar et al. 2015), and the lowest use of modern contraceptives among women in need (39 percent) (Campbell et al. 2015).

Contraceptive use is known to be affected by a number of factors, including socio‐demographic characteristics (age, residence, religion, educational and socioeconomic status), family characteristics and preferences (number of living children, desired family size), and family planning program exposure (Worku, Tessema, and Zeleke 2014). Conversely, women may choose not to use contraceptive methods because of fear of side effects, underestimation of pregnancy risk, partner's opposition, religious prohibition, and/or cost (Bellizzi et al. 2015). A review of the literature on factors influencing contraceptive use in sub‐Saharan Africa found the most common determinants to be fear of side effects, male partner disapproval, sociocultural norms around fertility, as well as women's education, employment, and communication with the male partner (Blackstone, Nwaozuru, and Iwelunmor 2017).

Marketing science uses a number of techniques to understand demand and distinguish between different types of potential users of a product or service (USAID and Reproductive Health Supplies Coalition 2009). In public health, analyses of demand and social marketing have been used in mass communication campaigns on HIV/AIDS and immunization, among other interventions (Grier and Bryant 2005; Noar et al. 2009). More recently, public health professionals have taken up these techniques more systematically as part of a “total‐market approach” to identify underserved populations and follow and understand trends in demand (USAID 2012). However, FP program planners rarely have access to detailed characterizations of demand that account for patterns of use and access, service preferences, and psycho‐social determinants of use (Chapman, Collumbien, and Karlyn 2006) as well as need, responsiveness to outreach and advertising, and accessibility of the client group (USAID and Reproductive Health Supplies Coalition 2009).

Existing analyses of FP demand tend to categorize clients based on demographic characteristics, for example ethnicity, age, or socioeconomic group (Patsika et al. 2009; PSI 2007, 2010; USAID 2011; Winfrey and Lacayo 2013); however, these variables are not necessarily the best predictors of actual behaviors, including FP use (Campo et al. 2012). While socio‐demographic variables can serve as an approximation when no other data exist, measures of attitudes (e.g., fertility desires, perception of social norms, perceived agency) and FP practices and behaviors (e.g., information seeking, discussion, and decision‐making) are preferable as they can yield a much more detailed understanding of fertility intentions and potential demand for contraception. These types of data can be used to construct attitudinal and/or behavioral (rather than demographic) segmentations, which can be powerful tools for understanding intent to change behaviors (Person et al. 2014; Yankelovich and Meer 2006). Findings can be used by program planners to create profiling tools to identify and target segments for improved communication, or by offering adapted service delivery.

Located in the West African Sahel, Niger is a low‐income country with the world's highest fertility rate (7.6 children per woman in 2012, increased from 7.1 in 2006) and a high maternal mortality ratio (estimated at 553 deaths per 100,000 live births in 2013) (DHS 2006, 2012; WHO 2015). Yet Nigeriens continue to desire even larger families: married women on average would like to have 9.2 children and married men 11.5 children (DHS 2012). In alignment with national goals to reduce annual population growth from 3.3 to 2.5 percent, the Nigerien Ministry of Public Health seeks in its 2012–2020 strategy to increase contraceptive prevalence from 16 percent in 2012 to 50 percent in 2020, and reduce total fertility from 7.6 to 5 children per woman (MSP 2012).

This article provides a case study of a demand analysis of FP in Niger, based on research performed in partnership with the Ministry of Public Health over a year beginning November 2013. The William and Flora Hewlett Foundation supported Hope Consulting (now part of Camber Collective) to conduct research and provide strategic recommendations to support and inform Nigerien FP programming. We first describe the research methodology and then present results of the national survey and resulting segmentation of Nigerien women with respect to FP demand. Finally we explore how this approach can be used to reach national FP goals in countries like Niger that seek to increase FP demand and enact effective FP policies.

## METHODS

Our study in Niger used a phased methodology based on techniques from commercial marketing science, as described below (Yankelovich and Meer 2006), with the objective of producing results relevant for achieving Nigerien national FP goals by informing communication campaigns, product innovation, pricing, and choice of distribution channels.

Data collection was preceded by consultation with local stakeholders and a literature review drawing from a range of disciplines to understand Nigerien women's fertility management context, including social anthropology, behavioral economics, cultural and health psychology, demography, and gender studies; as well as marketing science and the psychology of consumer behavior. Exploratory qualitative research was then conducted in November 2013, focusing on the Nigerien sociocultural and FP context and including focus group discussions, in‐depth interviews with FP providers, and direct observation of FP consultations with urban, peri‐urban, and rural women in and outside Niamey, Tahoua, and Zinder.

We used findings to inform the design of a representative survey of women of reproductive age (15–49 years) on FP in accessible regions of Niger (Table 1). Questionnaire development was inspired in part by the Integrated Behavioral Model, which posits intent or decision to perform a behavior as the most important predictor of whether the behavior will occur, to ensure inclusion of the broad range of drivers influencing an individual's intent to change their behavior, and was designed with Nigerien collaborators and subject area experts. The questionnaire was refined during the training workshop for data collectors and piloted over three days in greater Niamey. A sample size of 1,848 women was estimated based on a 99 percent confidence level and 3 percent margin of error with maximum variability; we rounded up to ultimately include 2,004 women to ensure our sample was sufficiently powered to detect differences. The survey was administered in April‐May 2014 using a two‐stage sampling methodology adapted from Demographic and Health Surveys (DHS) and MICS (i.e., random sampling of enumeration areas from the latest Niger census, stratified by region and urban/rural residence, with random walks to select households (ICF International 2012; Unicef 2005), except in Diffa, Tillabéri, and the rural parts of Agadez region, where data collection was precluded for security reasons. The number of enumeration areas in each region was based on population size as reported in the 2012 census data.

**Table 1:** Characteristics of a sample of women of reproductive age

	N (%)
**Age**	
15–19	355 (18)
20–29	803 (40)
30–39	577 (29)
40–49	269 (13)
**Highest education level**	
None	1,221 (61)
Primary	471 (24)
Secondary or higher	312 (16)
**Place of residence**	
Urban or peri‐urban	429 (21)
Rural	1,575 (79)
**Region**	
Agadez	36 (2)
Diffa	0 (0)
Maradi	522 (26)
Dosso	354 (18)
Tahoua	455 (23)
Zinder	476 (24)
Niamey	161 (8)
Tillabéri	(0)
**Religion**	
Muslim	1,981 (99)
Other	23 (1)
**Marital status**	
Married or living with partner	1,767 (88)
Divorced, separated, widowed	79 (4)
Single, never married	158 (8)
**Reproductive health characteristics**	
Pregnant at time of survey	328 (16)
Age at first sex <15 years	384 (25.8)
Ideal number of children (mean)	9.4
**Parity**	
0 children	297 (15)
1–2	527 (26)
3–4	516 (26)
5–6	376 (19)
7+	288 (14)
**Distance from nearest health center**	
0 km	895 (56.8)
1–4 km	325 (20.6)
5+ km	355 (22.5)
**TOTAL**	**2,004 (100)**

NOTE: Percentages may not add up due to rounding.

Questions focused on women's demographic and household characteristics; attitudes about spacing, limiting, and ideal family size; perception of social norms; characteristics sought in contraception; trusted advisers on sexual health and care‐seeking behaviors; relationship dynamics with husband/partner; and attitudes about religion, decision‐making, and self‐efficacy. When discussing FP methods, we first asked women if they were aware of any ways to delay or avoid pregnancy, collected spontaneous awareness of the different methods, then assisted awareness of methods that had not been mentioned spontaneously. For every method the respondent was aware of, we asked about consideration of use, trial of method, use of method within the last 30 days, and consistency of use. Written consent was required, with parental consent for unmarried minors.

We then reduced the sample to include only women whose memory and/or experience of fertility management was likely to be both recent and salient, as these conditions facilitate information retrieval and thus accuracy in reporting in survey methodology (Krosnick and Presser 2009). For this reason, we excluded women who said they were unable to become pregnant (N = 371). Separately, we also excluded women whose pattern of responses was deemed unreliable (notably those answering “yes” to almost every question, N = 46, not mutually exclusive with the previous group). We did include women who said “becoming pregnant now would not be a problem” (N = 524) since women who are passive about pregnancy may still manage their fertility or want or need FP services. From over 200 variables, we selected approximately 67 likely to be of high salience based on coherence with qualitative findings and correlation to acceptance and use of modern and traditional FP using chi‐2 tests (Table 2). We then used an iterative process to review two dozen possible segmentations with 5–8 classes each using Latent Gold software (version 4.5). We opted for latent class analysis because these models 1) are less subject to biases occurring when data does not conform to traditional modeling assumptions (linearity, normality, homogeneity), 2) accommodate mixed‐scale types (nominal, ordinal, etc.), and 3) can simultaneously assess relationships based on identification of classes and covariates (Magidson and Vermunt 2005). We used Bayesian Information Criterion (BIC) values to determine the ideal number of subgroups and Wald statistics to ascertain whether resulting segmentations produced substantive patterns, sometimes recoding variables to highlight patterns differentiating subgroups (Magidson and Vermunt 2002; Magidson and Vermunt 2004; Vermunt and Magidson 2002).

**Table 2:** Variables initially deemed to be of high salience for the family planning segmentation

Category	Survey question(s)^a^	Possible responses
**Reproductive attitudes**	Attitudes on reproduction:	
	1. *Acceptance of spacing*: Do you think it's acceptable for a couple to try to space the births of their children?	Yes / No / Don't know
	2. *Acceptance of limiting*: Do you think it's acceptable for a couple to try to limit the amount of children they have?	Yes / No / Don't know
	3. *Acceptance of the use of modern methods*: Do you think it's acceptable for a couple to use methods such as condoms, the pill, the morning‐after pill, and/or injectables to delay or avoid pregnancy?	Yes / No / Don't know
	4. *“If a woman still has a child on her back, she shouldn't get pregnant”*	Agree / Disagree / Don't know
	5. “If your oldest daughter is pregnant, you shouldn't be pregnant too”	Agree / Disagree / Don't know
	6. “Your husband will hate you if you have a child every year”	Agree / Disagree / Don't know
	Attitudes about autonomy:	
	7. “You can't be too young when you have your first child”	Agree / Disagree / Don't know
	8. “I don't like the government telling me how to manage my family”	Agree / Disagree / Don't know
	9. *“When it comes to decisions about my health, it's my choice”*	Agree / Disagree / Don't know
	10. “Sex is better when I don't have to worry about getting pregnant”	Agree / Disagree / Don't know
	Attitudes about religion:	
	11. *“It's a sin to use contraception”*	Agree / Disagree / Don't know
**Proactivity**	12. *Tried to get information on contraception*	By myself / With husband or partner / Not involved
	13. *Trust health‐care workers for FP info*	Yes / No / Don't know
**Social influence**	14–16. *How do you think the women in your community feel / would feel about other women using one of these (modern) methods?*	Acceptable / Not acceptable / Don't know (for 3 methods)
	17–19. How do you think your religious leaders feel / would feel about women using one of these (modern) methods?	Acceptable / Not acceptable / Don't know (for 3 methods)
	20. Perceived privacy of contraceptive consultations: “People know when a women talks to health workers about her sex life.”	Agree / Disagree / Don't know
**Method preferences**	Factors when deciding to use contraception:	
	21. *Geographical access/ proximity to health center*	Important / Not important / Don't know
	22. *Lack of opposition from husband, imams, friends, and family*	Important / Not important / Don't know
	23. *Husband's permission*	Important / Not important / Don't know
	Factors when choosing a contraceptive method:	
	24. *Fertility immediately after discontinuation*	Important / Not important / Don't know
	25. *Ability to stop at any moment*	Important / Not important / Don't know
	26. *Method is “natural”* 1	Important / Not important / Don't know
	27. *Simplicity of use*	Important / Not important / Don't know
**Behaviors and FP usage**	28. *Attended information session or had consultation on contraception*	Yes / No / Don't know
	29–41. *Awareness of any method (traditional* 1 *and modern* 2)	Spontaneous / Prompted / No (for 13 methods)
	42–54. *Consideration of specific traditional and modern methods*	Yes / No / Don't know (for 13 methods)
	55–67. *Use of specific traditional and modern methods*	Yes / No / Don't know (for 13 methods)

^a^Variables in italics were retained for use in the final segmentation. ^1^“Natural” or “traditional” methods were defined as not containing any chemicals or foreign substances/device, and include abstinence, lactational amenorrhea (LA), rhythm method, amulets/grigris, withdrawal method. ^2^Modern methods include contraceptive pill, male/female condom, injectable contraception, IUD, implants, morning‐after pill, male/female sterilization.

A 5‐class solution using 29 variables was retained on the basis of two main criteria: 1) high discrimination between subgroups, and 2) programmatic usefulness to in‐country actors based on intelligibility and practical implications. We discussed the analysis in consultation with technical experts, Nigerien collaborators, and national stakeholders during a two‐day workshop in Niamey in November 2014 attended by over 70 participants.

This research was approved by Niger's national ethics committee and Ministry of Higher Education and Scientific Research.

## RESULTS

Respondents to the survey were 2,004 women aged 15–49 years (Table 1), with an estimated response rate of 95 percent. Response rate did not differ significantly in urban and rural areas or by region. Compared to the 2012 DHS sample of women (N = 11,160), our overall sample was similar in age, place of residence, and marital status, although more educated (39.1 percent of women had some education compared to 19.9 percent in DHS) and less likely to have 7 or more children (14.4 percent compared to 24.6 percent). The vast majority of women in our sample (98.9 percent) were Muslim; most (77.4 percent) lived within 5 kilometers (3 miles) of a health center, and nearly half (47.8 percent) had seen a health worker for their own health in the past three months. Sixteen percent were pregnant at the time of the survey.

**Figure 1:**
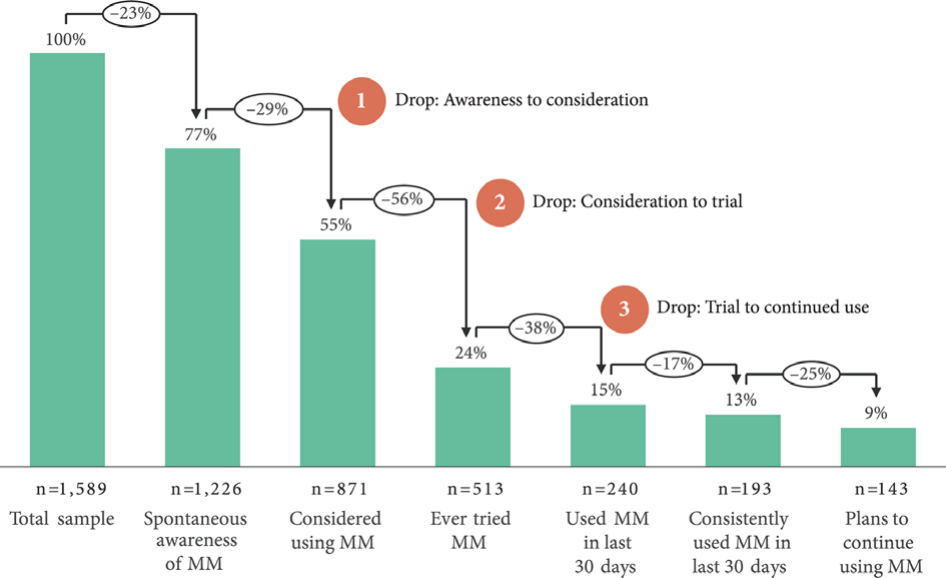
Demand for modern methods of contraception in a subsample of women in Niger (N = 1,589) NOTE: MM = Modern methods of contraception (contraceptive pill, male/female condom, injectable, IUD, implant, morning‐after pill, male/female sterilization).

After removing women who were not fertile, in the subsample of women used for the demand analysis (N = 1,589), FP knowledge was moderately high and similar to the overall sample (77 percent of women could spontaneously name at least one modern method). However a continuum of FP demand (Figure 1) shows large and specific drop‐offs between awareness of modern methods, consideration, and consistent use. Twenty‐nine percent of women who were aware of modern methods said they did not consider using any modern method. Among those who had considered using one, only 44 percent said they had ever tried one. Of those who had tried one, only 62 percent had used one in the past 30 days. However, among women who had used a modern method in the last 30 days (15 percent of women), large percentages used it consistently and planned to continue use.

The FP segmentation draws on factors hypothesized to guide women in their steps from knowledge to trial and current use, with the main differences between the five subgroups presented in Table 3. Demographic covariates, while not used to create the subgroups, also varied across them (Table 4) and were used to situate the demand profiles of subgroups. Among the variables used to build the segmentation, three were found to be most important in establishing differences among subgroups based on Wald statistics. These had to do with general preferences for characteristics of an FP method, specifically whether women said it was important that 1) when they stopped using the method, they were fertile right away, 2) it was easy to stop at any moment, and 3) the method was “natural.” Other variables strongly contributing to differentiating subgroups were: acceptance of spacing; preference for a simple method; awareness and use of modern methods; the number of times a woman tried to get information on FP; acceptance of limiting; perceived opposition from friends, family, and husbands; beliefs about the number of women using contraception in their community; and the respondents’ involvement in health decision‐making. Geographic access and cost of contraception were rarely mentioned as determining factors, as FP methods are widely available at no cost in government health centers.

**Table 3:** Segmentation of women's FP demand in Niger

	Group 1	Group 2	Group 3	Group 4	Group 5	Overall sample
**Proportion of total** – N (%)	248 (16)	441 (28)	164 (10)	294 (19)	442 (28)	1,589 (100)
**Reproductive attitudes** – N (% of subgroup)						
1. Accept spacing	228 (92)**	415 (94)**	144 (88)**	254 (86)**	116 (26)**	1,157 (73)
2. Accept modern methods^1^	196 (79)**	365 (83)**	106 (65)**	130 (44)**	78 (18)**	875 (55)
3. Accept limiting	129 (52)**	177 (40)**	12 (7)**	79 (27)	44 (10)**	441 (28)
4. “A woman with a child on her back shouldn't get pregnant”	169 (68)	353 (80)**	156 (95)**	213 (72)	284 (64)**	1,175 (74)
5. “Decisions about my health are my choice”	103 (42)	195 (44)	127 (77)**	84 (29)**	170 (39)*	679 (43)
6. “It's a sin to use contraception”	108 (44)	143 (32)**	45 (27)**	156 (53)**	215 (49)**	667 (42)
**Proactivity** – N (% of subgroup)						
7. Involved in FP decisions with husband/partner	156 (63)**	266 (60)**	104 (63)**	108 (37)**	68 (40)**	702 (53)
8. Has tried to get more info on contraception	204 (82)**	341 (77)**	164 (18)**	36 (12)**	39 (23)**	784 (49)
9. Trust health‐care workers the most to discuss FP	83 (33)**	202 (46)**	30 (18)**	51 (21)**	49 (28)**	415 (32)
**Social influence** – N (% of sub‐group agreeing)						
10. Some/many women use condoms	126 (51)**	152 (35)**	9 (6)**	37 (13)**	37 (22)	361 (27)
11. Some/many women use pills or injectables	229 (92)**	402 (91)**	132 (81)	236 (80)**	139 (81)*	1,338 (86)
12. Some/many women use implants, IUDs, sterilization	140 (57)**	225 (51)**	49 (30)**	95 (32)**	83 (43)	592 (45)
**Method preferences** – N (% subgroup saying it's important)						
13. Geographical access to health facility	200 (81)*	349 (79)*	125 (76)	228 (78)	90 (52)**	992 (75)
14. Husband not opposed	164 (66)*	236 (54)**	122 (74)**	213 (72)**	66 (38)**	801 (61)
15. Friends and family not opposed	144 (58)**	138 (31)**	62 (38)	164 (56)**	50 (29)**	558 (42)
16. Don't need husband's permission	129 (52)**	192 (44)	50 (31)**	116 (40)	49 (29)**	536 (41)
17. Ability to stop at any moment	220 (89)**	376 (85)	141 (86)**	197 (67)**	0 (0)**	934 (71)
18. Method won't impact my fertility	193 (78)**	306 (69)	142 (87)**	199 (68)	74 (43)**	914 (69)
19. Method is natural	248 (100)**	0 (0)**	110 (67)**	245 (83)**	0 (0)**	603 (46)
20. Simple to use	230 (93)*	420 (95)**	155 (95)*	258 (88)	114 (66)**	1,177 (89)
**Behaviors and FP usage** a – N (% of subgroup)						
21. Attended an info session/had an FP consultation	81 (33)**	143 (32)**	25 (15)**	48 (16)**	47 (11)**	344 (22)
22. Awareness of one or more traditional method^2^	233 (94)**	427 (97)**	154 (94)**	427 (97)**	163 (37)**	1,404 (79)
23. Awareness of one or more modern method^1^	248 (100)**	440 (100)**	164 (100)**	293 (100)**	172 (39)**	1,317 (83)
24. Consideration of one or more traditional method^2^	55 (22)**	232 (53)**	122 (41)**	92 (56)**	69 (16)**	570 (36)
25. Consideration of one or more modern method^1^	211 (85)**	356 (81)**	99 (60)**	111 (38)**	94 (21)**	871 (55)
26. Current traditional method use^2^	22 (9)**	101 (23)**	52 (32)**	54 (18)	19 (4)**	248 (16)
27. Main traditional method used^3^	n/a	LA (14), abs. (9)	LA (28)	LA (12)	n/a	n/a
28. Current modern method use^1^	98 (40)**	120 (27)**	4 (2)**	8 (3)**	10 (2)**	240 (15)
29. Main modern method(s) used^3^	Pill (27),	Pill (22),	n/a	n/a	n/a	n/a
	injectable (13)	injectable (11)				

^*^Significant at p < 0.05; ^**^p < 0.01. ^a^Among women who are sexually active and premenopausal. ^1^Modern methods = Contraceptive pill, male/female condom, injectable contraception, IUD, implants, morning‐after pill, male/female sterilization. ^2^Traditional methods = Abstinence, lactational amenorrhea (LA), rhythm method, amulets/grigris, withdrawal method. ^3^Among women who have used one or more methods in the past 30 days. n/a = Not applicable.

**Table 4:** Demographic covariates of subgroups

	Group 1	Group 2	Group 3	Group 4	Group 5	Overall sample
**N (% of sample)**	248 (16)	441 (28)	164 (10)	294 (19)	442 (28)	1,589 (100)
**Average age** – years	28	28**	26	27	25**	27
**Secondary or higher education** – N (%)	57 (23)**	69 (16)	18 (11)**	50 (17)	58 (13)**	252 (16)
**Higher wealth index** **a** – N (%)	128 (51)**	203 (46)**	41 (25)**	96 (32)**	103 (23)**	571 (35)
**Urban residence** – N (%)	84 (34)**	97 (22)*	18 (11)**	50 (17)*	73 (17)*	322 (20)
**Married** – N (%)	71 (85)	93 (96)**	15 (83)	36 (72)**	60 (82)	275 (85)
Waited until age 18 to marry	47 (67)**	55 (66)	7 (47)	16 (48)	19(33)**	144 (54)
**Parity** – N (%)						
0 children	11 (13)*	8 (8)**	5 (28)	20 (40)**	23 (32)*	67 (21)
1–4	53 (63)	68 (70)**	9 (50)	19 (38)**	35 (48)*	184 (57)
5+	20 (24)	21 (22)*	4 (22)	11 (22)	15 (21)*	71 (22)
**Considers herself “very religious”** – N (%)	93 (38)**	72 (17)**	32 (20)**	83 (28)**	132 (30)**	412 (26)
**Earned money independently** – N (%)	31 (37)	37 (38)*	3 (17)	13 (26)	10 (14)**	94 (29)
**Visited health center in last month** – N (%)	127 (51)	236 (54)*	79 (48)	133 (45)*	211 (48)	786 (49)
**Being pregnant today would be a problem** – N (%)	88 (43)**	124 (36)**	58 (44)**	138 (59)**	116 (34)**	524 (42)

^*^Significant at p < 0.05; ^**^p < 0.01. ^a^Based on a weighted count of household and other material goods.

NOTE: Numbers and percentages in the Overall Sample column may not correspond to the full sample of 1,589, because some study questions had fewer numbers of women responding.

Women in Group 1 (16 percent of the sample) have the highest rate of modern method use and are the only subgroup to accept using FP for limiting births. Group 2 women (28 percent) also tend to be more approving of FP; however, they use traditional as well as modern methods to meet their demand and trust health‐care workers to get information on FP more than any other group. Women in Group 3 (10 percent) are somewhat less accepting of modern methods and much less accepting of limiting births using FP, although they accept spacing. They are the greatest users of traditional methods of FP in the sample. Women in Group 4 (19 percent) are much less accepting of modern methods compared to the previous subgroups, and are most concerned using FP will incur social disapproval. Finally, Women in Group 5 are by far the least informed about FP, do not use any method, and are highly mistrustful of it.

### Group 1: Women Who Accept Limiting

These women are significantly more likely than the overall sample to accept birth spacing (92 percent compared to 73 percent of all women, p < 0.01) and the use of modern methods (79 percent compared to 53 percent, p < 0.01). They are the only subgroup in which the majority of women accept the use of contraception to limit the number of births (52 percent versus 28 percent, p < 0.01). They are much more likely to have used a modern method of contraception in the last 30 days (40 percent versus 15 percent average, p < 0.01), with most preferring the contraceptive pill or injectable, and are most likely to believe other women in their community also use modern methods. These women are also among the least likely to use a traditional method. While women in this group are more likely to identify themselves as “very religious” compared to any other subgroup (38 percent versus 26 percent average, p < 0.01), they are no more likely to agree with the statement “It's a sin to use contraception” (44 percent versus 42 percent average, p > 0.05). Group 1 women are somewhat more urban, better‐educated, and wealthy than the rest of the sample, with 34 percent living in urban areas compared to 20 percent in the overall sample and nearly half scoring high on a weighted wealth index compared to 35 percent overall (p < 0.01); they are also much more likely to have waited until age 18 to get married (67 percent versus 54 percent, p < 0.01).

### Group 2: Women Who Trust FP and the Health System

Women in this group are defined by high acceptance of FP for birth spacing, and proactive behaviors in seeking out FP information and services. Along with women in Group 1, they are the only subgroup to have used a modern method of contraception within the last 30 days at any significant rate (27 percent versus 15 percent on average and ≤4 percent for the three remaining subgroups, p < 0.01), also preferring the contraceptive pill or injectables. Unlike Group 1 women, however, they also use traditional methods (mainly lactational amenorrhea or abstinence), with nearly a quarter using such a method within the last month (p < 0.01). These women nearly universally accept the use of contraception for birth spacing (94 percent versus 73 percent average, p < 0.01). They are the subgroup most likely to have been to a health‐care center in the last month (54 percent versus 49 percent average, p < 0.05) and are most likely among women to trust health‐care workers to discuss FP more than anyone else (46 percent versus 33 percent average, p < 0.01). They are less likely than most subgroups to be concerned about opposition from husbands, imams, friends, or family (p < 0.01) and are significantly less likely than average to believe that contraception is a sin. Slightly older, more urban, and wealthy compared to the sample average, they are also most likely to be married (96 percent compared to 85 percent average, p < 0.01).

### Group 3: Women with a Customary View of Reproduction

Women in this subgroup hold traditional beliefs about when a woman should become pregnant and a strong sense of autonomy over contraceptive decision‐making, in conjunction with their husbands. These women have among the lowest rate of use of modern methods (2 percent versus 15 percent average, p < 0.01) and are least likely to report that other women in their community use any method of modern contraception. At the same time, they have the highest rate of traditional method use (32 percent versus 16 percent, p < 0.01), mainly lactational amenorrhea. Their traditional beliefs around FP are indicated by a rejection of birth limiting (accepted by only 7 percent versus 28 percent average, p < 0.01); they are also most likely to agree with the statement “If a woman still has a child on her back, she shouldn't get pregnant” (95 percent, p < 0.01), which is a traditional way of expressing support for longer periods of child spacing. These women also express the view that women are in charge of their reproductive lives and are the most likely subgroup to say “When it comes to decisions about my health, it's my choice” (77 percent versus 43 percent average, p < 0.01); they are also more likely to be involved in contraceptive decision‐making with their husband or partner (63 percent versus 53 percent average, p < 0.01). This subgroup strongly agrees more than any other that “I do not want the government telling me how to manage my family” (52 percent versus 31 percent average, data not shown). Consistent with their use of traditional methods, this subgroup prefers methods that are free and easy to access, have no side effects, are approved by their husbands, and, especially, do not impact subsequent fertility (87 percent, p < 0.01). Demographically, these women are less likely than average to have secondary education (11 percent versus 16 percent average), are less wealthy (25 percent high wealth index score versus 35 percent average), and are more rural (11 percent live in urban areas compared to 20 percent on average) (p < 0.01).

### Group 4: Women Who Fear Social Condemnation

Women in Group 4 are primarily concerned with social norms, religious rules, and the approval of their families and communities when it comes to FP. Women in this subgroup are less likely than average to accept the use of modern methods (44 percent versus 55 percent average, p < 0.01), have used them in the past month (3 percent versus 15 percent, p < 0.01), or believe that other women in the community use any type (p < 0.01). While Group 1 women also consider themselves very religious, Group 4 women hold relatively stricter religious beliefs such as believing that Islamic teachings are not open for interpretation (93 percent, data not shown). They are most likely of all subgroups to agree with the statement “It's a sin to use contraception” (53 percent versus 42 percent average, p < 0.01). They highly value the permission of their husbands, other women, and imams, and tend to trust friends and family most when discussing FP. At the same time, these women are least likely to be involved in contraceptive decision‐making (37 percent versus 53 percent average, p < 0.01) and to agree with the statement “When it comes to decisions about my health, it's my choice” (29 percent versus 43 percent average, p < 0.01). Perhaps given their perception of societal and religious disapproval of FP, this subgroup appears highly disinterested in learning more: only 12 percent had tried to get more information on FP versus 49 percent average (p < 0.01) and 16 percent have had an FP consultation versus 22 percent average (p < 0.01). However, compared to women in Group 5, women in Group 4 still value spacing quite a bit, and use traditional methods at average rates. This subgroup is slightly less urban than the others (17 percent compared to 20 percent average, p < 0.05) but otherwise does not stand out demographically.

### Group 5: Women Who Think FP Is Not Their Concern

Women in this subgroup are characterized by a notable lack of knowledge and acceptance of FP, and low autonomy in decision‐making. These women have the lowest average age (25 years, compared to 27 years average, p < 0.01); they are also more likely to live in a rural area and have lower‐than‐average wealth and education. They are the only subgroup to systematically reject FP for both spacing (26 percent accepted versus 73 percent average, p < 0.01) and limiting (10 percent versus 28 percent average, p < 0.01); they also have the lowest rates of acceptance of modern methods (18 percent versus 55 percent average, p < 0.01) and the lowest current use of modern and traditional methods (2 percent and 4 percent respectively, p < 0.01). They are the subgroup least interested in learning more about FP and are less likely than average to agree that *any* factor was important when choosing a contraceptive (p < 0.01 for all factors). These women also have low autonomy, with lower‐than‐average agreement with the statement “When it comes to decisions about my health, it's my choice” (39 percent versus 43 percent average, p < 0.05). More so than all other subgroups, Group 5 women evinced both ignorance of and rejection of FP. They also have the lowest unmet need as measured by the variable “being pregnant today would be a problem” (34 percent versus 42 percent average, p < 0.01).

## DISCUSSION

Unmet need for FP in low‐ and middle‐income countries rose to an estimated 962 million women in 2015 and with it preventable pregnancy‐related deaths and morbidity, as well as increased risk of preterm births and resulting complications (Alkema et al. 2013; Streatfield et al. 2014). In a “quality of services” approach, promoting “access” to FP means not only promoting affordability and geographic proximity of services but also providing women with accurate information about risks and benefits, as well as “psychosocial access” via the acceptability of contraception and associated services (Cleland, Harbison, and Shah 2014; Machiyama and Cleland 2014). Demand generation interventions (counseling, education activities, financial incentives) have been found to be positively associated with increases in current use of modern contraceptive methods, with a recent meta‐analysis of nine studies including 14,235 participants showing a pooled OR of 1.57 (95% CI: 1.46‐1.69, p < 0.01, 9 studies) (Belaid et al. 2016). However programmatic interventions have often focused on supply‐side approaches, leaving social and behavior change (SBC) campaigns and demand generation as an afterthought. Significant progress has been made in improving geographic and financial access to FP in many countries; however, preference for large families continues to drive low contraceptive prevalence rates especially in sub‐Saharan Africa (Cleland, Harbison, and Shah 2014; Canning, Raja, and Yazbeck 2015). New approaches to FP programming are required, incorporating an understanding of women's needs, attitudes, and desires so as to improve psychosocial access and offer services and information that speak to women's fertility desires and contraceptive preferences.

Our research aimed to identify the most relevant attitudinal factors driving FP use and to group women according to their FP attitudes and practices. Women in five subgroups displayed varying degrees of motivation and autonomy; preferred channels for learning about FP; preferred methods and method characteristics; and acceptance of or opposition to FP (Table 5). The first two subgroups, Groups 1 and 2, together representing nearly half of the women in the sample, may be relatively well‐served by traditional approaches to providing FP, as these women are not opposed to modern or traditional methods, are adequately informed, do not fear social stigmatization for using FP, and proactively reach out to health services. Interestingly, Group 1 women were distinguished by a high degree of religiosity, perhaps reflecting a need to show they uphold moral values despite being the only group to accept FP for the purposes of limiting (Moumouni 2016). Both Groups 1 and 2 trust health‐care workers and thus can be reached through the health‐care system and served by ensuring steady supplies of modern methods and well‐informed health workers. However, roughly half of Group 2 women rely heavily on traditional methods (abstinence and lactational amenorrhea). Service delivery and messages are needed to help these women identify and adopt strategies to decrease their exposure to pregnancy, such as ensuring they are protected when they resume sexual activity or when one of the three criteria for lactational amenorrhea coverage is not met (exclusive breastfeeding, child is less than 6 months, and menses have not yet returned), as well as to propose additional effective methods.

**Table 5:** Summary of subgroup profiles and implications for programming

	Group 1	Group 2	Group 3	Group 4	Group 5
**Percent of sample (%)**	16	28	10	19	28
**FP awareness and knowledge**	**++**	**++**	**++**	**++**	**−**
**Acceptance of FP**	**++**	**++**	**+**	**−**	**− −**
**Use of modern methods**	**++**	**+**	**−**	**−**	**−**
**Proactivity in seeking FP information**	**+**	**++**	**+**	**−**	**−**
**Family planning need** **1**	**+**	**+**	**+**	**++**	**−**
**Considerations when targeting with interventions**	Small subgroup relative to population Likely to access FP already Self‐identify as virtuous Low resonance with other groups in terms of potential messages Can serve as a model for other subgroups	Large subgroup High potential for uptake (similar to Group 1 but less educated) Strong interest in personal and family health Trusts health‐care workers Opportunity to increase spacing, limiting, and modern method use	Smallest subgroup Interested mainly in traditional methods Makes her own decisions, with husband's input Potential to convert to modern methods	High need Some interest in traditional methods for spacing Not autonomous Opinion of others is very important, linked to FP resistance Approval of spacing may be best entry point Work with community and husbands is key	Large subgroup Little knowledge but strongly resistant to FP Not autonomous Opportunity for growth if educated Potential to direct into other subgroups as they grow up

^1^Based on percent of women who answered “yes” to the question “Would it be a problem if you became pregnant today?”

FP = Family planning. + = High. ++ = Very high. − = Low. − − = Very low.

Our results additionally suggest that the other three subgroups—Groups 3, 4, and 5, making up 57 percent of the sample—are unlikely to be adequately reached solely via the health system at this time. Women in these subgroups were significantly less accepting of contraception of any kind, and less proactive about seeking out information. These subgroups would likely benefit from well‐designed, sensitive, and ethically informed outreach, SBC campaigns, and community‐based interventions. Among these three groups, it is likely that Group 4 would be a difficult group to engage and encourage a change in FP behavior, as their barriers to FP use are grounded in perceptions of social and religious norms that would take longer to address. On the other hand, Group 5 women (28 percent of the sample) could merit special attention, because of these women's youth, lack of knowledge of FP, and low level of pregnancy avoidance needs. Messages for them could include more general encouragement to learn about the world and consider life choices. (Implementation toolkit and programmatic guidance are available, in English and French: http://www.cambercollective.com/fpniger/.) Group 3 women's practices stem from customary beliefs, which value birth spacing (these women were most likely to believe “A woman with a baby on her back shouldn't get pregnant) and women's autonomy (they were also most likely to believe “Decisions about my health are my choice”), while also promoting high fertility ideals (they were least accepting of all groups of birth limiting). As such, Group 3 women may be best reached via appeals to traditional values that are sensitive to and engage with positive images of fertility and large families. Different arguments and approaches are needed to address these diverse types of “opposition” to FP.

Taken together, these subgroups of women illustrate the diverse obstacles to promoting psychosocial access to FP in this high‐fertility context, including dimensions of knowledge, accessibility, trust, and acceptability. Group 5 paints the portrait of those women who do not know yet about modern contraception and yet mistrust it; whereas women in Groups 3 and 4 know about modern contraception but do not contemplate using it, although for different reasons. Group 2 women intend to use modern contraception, but many end up using less effective traditional methods instead (likely as a strategy of risk avoidance with respect to partners and societal norms). Women in Group 1 are alone in having navigated these obstacles to more or less consistently use modern contraception.

This study has some limitations. Primary data collection was geographically restricted for security reasons, causing us to limit or omit sampling from the country's northern and eastern regions (Diffa and Agadez) and in Tillabéri region. This could explain some sample characteristics, such as the higher education levels compared to DHS. We attempted to mitigate for this bias by oversampling from neighboring regions and ensuring rural areas were fully represented in the final sample. We were unable to classify women by exposure to pregnancy risk (sexual activity in the last month, menses have not returned since last childbirth) as these variables were either not included in data collection or were considered potentially unreliable. Finally, a major limitation to our study is the lack of inclusion of men in our analysis, despite their strong role in FP decision‐making for many of the subgroups. Indeed engaging men as partners in FP discussions has been shown to lead to improved reproductive health outcomes, while also meeting men's own reproductive health needs (Hardee, Croce‐Galis, and Gay 2016). While we chose to focus on women for this exercise, in recognition of this limitation we are currently proceeding with a similar segmentation for men in Niger, with plans to triangulate between findings to further explore the relational nature of FP decision‐making.

This demand analysis was presented at a national workshop in November 2014 and has since been used in a number of ways to support FP programming in Niger and neighboring countries. A toolkit was created for each subgroup with profiles, programmatic guidance, and sample messages, and was used by the Nigerien national NGO Animas Sutura to design improved FP counseling services. A simple profiling tool containing 12 questions was developed to help service providers quickly categorize women by subgroup during a screening process. In a pilot test in 12 health centers in Maradi, exit surveys showed women receiving counseling that was adapted to their subgroup profile subsequently had higher awareness and use of modern methods than women receiving standard counseling (data available upon request). Health‐care workers said the adapted approach to counseling saved them time and improved their ability to connect with clients. The demand analysis is also being used in an SBC communication strategy designed by the international NGO EngenderHealth in Niger, targeting women in Groups 2 and 5 with communication approaches adapted to their profiles. Data from the Niger study have been used by EngenderHealth to design FP messages in Burkina Faso and Togo, focusing on barriers and opportunities to FP use by segment, however data and results issuing from these efforts are not yet available.

Preliminary evidence suggests that policymakers and program officers can use segmentations to target interventions and stimulate demand directly and indirectly (USAID and Reproductive Health Supplies Coalition 2009). FP stakeholders have expressed interest in a “total market approach” to FP, including demand analyses, in diverse national contexts (Drake et al. 2011; Drake et al. 2010), as well as in our study in Niger. Such demand‐centered approaches are likely to be particularly salient in contexts where women already have good access to FP and sufficient autonomy to act on their FP attitudes and desires. However much more research is needed to determine the effectiveness of segmentations in promoting psychosocial access to FP and supporting FP programming, as well as their cost‐effectiveness in low‐resource contexts such as Niger, given the not‐insubstantial costs of periodic data collection (Chapman et al. 2006). Nonetheless US$30 billion per year is invested in such techniques by private companies worldwide, including in Africa, suggesting they can produce results and may warrant further investigation (ESOMAR 2011). Such initiatives must not overshadow efforts to ensure women's autonomy and agency at the level of families, health systems, and societies, via mechanisms including girls’ education (including comprehensive sexuality education), female labor force participation, and the adoption of legal frameworks supporting gender equality. In contexts like Niger, where only a small fraction of the female population has access to even primary education, the role of education is likely to be particularly salient given its established link with FP use.
